# Recurrence of Japanese Encephalitis Epidemic in Wuhan, China, 2009–2010

**DOI:** 10.1371/journal.pone.0052687

**Published:** 2013-01-09

**Authors:** Quan Hu, Banghua Chen, Zerong Zhu, Junhua Tian, Yu Zhou, Xiaoyong Zhang, Xin Zheng

**Affiliations:** 1 Wuhan Centers for Disease Control and Prevention, Wuhan, China; 2 Division of Allergy and Immunology, Joseph Stokes Jr. Research Institute at The Children's Hospital of Philadelphia, Department of Pediatrics, Philadelphia, Pennsylvania, United States of America; 3 Institute of Virology, University Hospital of Essen, Essen, Germany; 4 Department of Infectious Disease, Union Hospital, Tongji Medical College, Huazhong University of Science and Technology, Wuhan, China; Utah State University, United States of America

## Abstract

**Background:**

Japanese encephalitis (JE) was once epidemic in most areas of China, including Wuhan, a city located in the central part of China. The incidence of JE dramatically decreased due to nationwide immunization with the live attenuated JE virus (JEV) vaccine, and no JE cases were reported during 2005–2008 in Wuhan. In 2009 and 2010, 31 JE cases reoccurred in this area. In this study, we investigated the causes of JE recurrence.

**Methods and Findings:**

All JE cases were laboratory-confirmed by detecting the JEV-specific IgM antibody with an IgM-capture enzyme-linked immunosorbent assay (ELISA). All patients were children between 2 months and 9 years of age with a median age of 2 years. Of the 31 cases, 9 had received one or two doses of the JEV vaccine, 11 had not been immunized previously with the JEV vaccine, and 11 had an unclear immunization history. Through reverse transcription polymerase chain reaction (RT-PCR), sequencing, and phylogenetic analysis, two new strains of JEV were isolated from *Culex tritaeniorhynchus* and identified as genotype 1 JEV, rather than genotype 3, which circulated in this area previously.

**Conclusions:**

Vaccine failure or missed vaccination may have caused JE recurrence. Local centers for disease control and prevention need to improve immunization coverage, and the efficacy of the JE vaccine needs to be reevaluated in a population at risk for disease.

## Introduction

Japanese encephalitis (JE) is an acute epidemic disease of the central nervous system caused by infection with the Japanese encephalitis virus (JEV), which primarily affects children and adolescents [1], [2]. It was recently estimated by the World Health Organization (WHO) that the annual case frequency of JE is 67,897 in JE-endemic areas, most of whom are children under 15 years old. The case mortality rate is 20–30%, and neurologic or psychiatric sequela occurs in 30–50% of survivors [2]–[5]. JE occurs throughout most of Asia and parts of the western Pacific [6], [7], [8].

Extensive JE vaccination programs have been implemented in JE endemic countries. Asian countries, like Japan and Korea, which have had major epidemics in the past, have already controlled JE through extensive JE vaccination programs. However, JE is still a life-threatening disease to people living in endemic areas in developing countries, mainly due to the difficulties of controlling the JE vector and amplifier [9]. In the 1990s, outbreaks were reported in Australia and on the island of Saipan. In both, mosquito vectors were believed to be involved [10], [11].

JEV is an arthropod-borne virus (arbovirus) that is transmitted in an enzootic cycle between mosquitoes and amplifying vertebrate hosts, mainly pigs and wading birds [12], [13], [14]. JEV is the most common pathogen leading to viral encephalitis in Asia. JEV strains have been divided into five genotypes, and genotypes 1 and 3 are distributed widely in Asia, including China, Japan, Korea, India, Vietnam, and the Philippines [15].

JE cases have been reported in most provinces of China except Xinjiang Uygur Autonomous, and Qinghai Province [1], [16]. Since an extensive JE vaccination program started for children in the 1970s, the number of JE cases has significantly decreased nationwide, from 174,932 cases of morbidity in 1971 to 5,097 cases in 2005 [16]. However, outbreaks still occur in some provinces, especially in the middle and western areas of China [16], [17]. Here, we report that 31 JE cases occurred from 2009 to 2010 in Wuhan, which is located in the central part of China and is the capital of Hubei Province.

**Figure 1:**
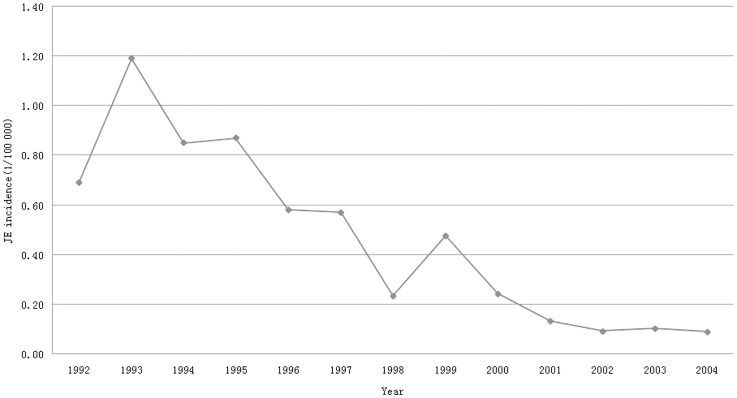
Incidence rate of Japanese encephalitis (JE) in Wuhan, China (1992–2004). From 1992 to 2004, the incidence rate of JE decreased in Wuhan, China.

In Wuhan, the incidence rate of JE dramatically decreased in the early 1990s (Figure 1), when a booster JE vaccination campaign began to immunize children under 15 years old in rural areas with live attenuated vaccine (SA14-14-2, manufactured by Chengdu Institute of Biological Products, China) in April every year at their own expense [18], [19]. Between 2005 and 2008, no JE cases were reported. In the present study, we collected epidemiological data from JE patients, piglets, and mosquitoes in the areas of confirmed JE cases to explore the possible causes for the recurrence of JE in the Wuhan area.

## Materials and Methods

### Ethics statement

All the experiments involving animals and humans were approved by the Ethics Committee of the Medical Research Council of Wuhan. Signed informed consents were obtained from parents prior to participation.

### Subjects

In 2009 and 2010, all suspected JE cases reported to the Chinese Disease Reporting Information System (CDRIS) in Wuhan were further investigated by the Wuhan Centers for Disease Control and Prevention (CDC) according to a WHO-recommended JE surveillance project [20]. Patients and caregivers were interviewed, medical records were checked, sera for JEV-specific antibody testing were collected, and other epidemiological data, such as history of JE vaccination (recorded date and vaccination dose were confirmed by reviewing immunization certificates) and travel history before disease onset, were collected. A suspected JE case is one that meets the clinical case definition for viral encephalitis syndrome, which is defined as a person with acute onset of fever and a change in mental status (including symptoms such as confusion, disorientation, coma, or inability to talk). A laboratory-confirmed case is one in which the JE virus-specific IgM antibody is detected from a single serum sample from the suspected case with an IgM-capture ELISA [20], which is the recommended method for laboratory confirmation of a JEV infection by the WHO. In addition, other serum testing was done to rule out infection due to a cross-reacting flavivirus-like dengue virus.

### Serological surveillance on piglets

Blood samples for serological studies were also collected from piglets (pigs aged less than 3 months) on a farm in Dongxihu District of Wuhan (near the areas of JE cases) from April to October 2009. The antibody response (i.e. IgM and IgG) to JEV was detected by JE Detect***™*** IgG/IgM antibody capture ELISA kits (Inbios International, Inc., Seattle, WA, USA).

### Mosquito monitoring

Mosquitoes were captured with light traps hung near pigpens close to JE case's houses. All mosquitoes captured were anesthetized by carbon dioxide and then grouped according to their type and sex. Mosquitoes were classified into three groups (Culex, Aedes, Anophele) and stored in tubes with 50–100 mosquitoes as a pool based on their classification. The tubes were kept frozen in a liquid nitrogen tank until virus isolation.

### Cell line and cell culture

BHK-21, a baby hamster kidney derived fibroblast cell line, obtained from the American Type Culture Collection (ATCC CCL10), was a gift kindly provided by the College of Veterinary Medicine, Huazhong Agricultural University, Wuhan, China. The BHK cells were cultured in Dulbecco's modified Eagle medium (DMEM) containing 2 mML-glutamine (Invitrogen, Grand Island, NY, USA) supplemented with 10% fetal bovine serum (Invitrogen, Melbourne, Australia), 100 units/ml penicillin, and 100 µg/ml streptomycin (Invitrogen, Grand Island, NY, USA) in T-25 flasks with 5% CO_2_ at 37°C.

### Virus isolation

Viruses were isolated as described previously [21], [22]. Briefly, 2 ml of DMEM was added into each tube containing the mosquito pool, followed by homogenizing the mixture in a glass homogenizer on ice. The homogenate was collected and centrifuged at 3,000 rpm for 15 min. The supernatant (200 µl) was filtered through a 0.2 µm syringe and added to BHK-21 cells in a 6-well culture plate containing 2% fetal bovine serum, 2 mM L-glutamine, 100 units/ml penicillin, and 100 g/ml streptomycin. After the cells were passaged at least three times, the supernatants were harvested after 5 days of culture.

### Virus identification

Total RNA was extracted from supernatants of infected BHK21 cells using an Rneasy Mini Kit (QIAGEN, Hilden, Germany). RT-PCR was performed with a one step RT-PCR kit (Qiagen, Hilden, Germany) to amplify a 1541 bp JE E gene fragment (JEEF). The primer sequences used for RT-PCR were as follows: forward primer JEEF 5′-GTCGCTCCGGCTTACAGTTT-3′ and reverse primer JEER 5′-GATGTCAATGGCACAGCCGT-3′. The RT-PCR conditions were as follows: cDNA synthesis at 50°C for 30 min, pre-denaturation at 94°C for 15 min, followed by 40 cycles of denaturation at 94°C for 45 s, annealing at 55°C for 45 s, and extension at 72°C for 90 s. The positive PCR products were sent to Sangon Biotech Co. Ltd. (Shanghai, China) for sequencing with bidirectional primer measuring communication.

### Multiple alignments and phylogenetic analysis

A total of 30 JEV strains, including two new JEV strains isolated from *Culex tritaeniorhynchus* near the JE cases' homes and the live attenuated vaccine virus strain SA14-14-2 were analyzed. In addition, Murray Valley encephalitis virus (MVEV) was analyzed as an out group strain. The nucleotide sequences of all strains were obtained from Genbank, along with their places of isolation, years of isolation, and accession numbers. The E gene and deduced amino acid sequences of all 30 strains were aligned with ClustalX 1.83, Mega software version 4.0, and the phylogeny tree was constructed using the neighbor-joining (NJ) method [23]. Constructed NJ trees were analyzed with bootstrap of 1000 replicates using Kimura's two-parameter method.

### Data management and analysis

Data for all cases, vectors, and contacts were entered into Excel 2007 spreadsheets (Microsoft, USA). The analysis was conducted by filter functions and statistical programs in this software. The Epi-Info (version 3.5.1, CDC, Atlanta, GA, USA) program was also used for statistical analysis. Double original data entries and computer printouts were repeatedly performed to verify data quality.

## Results

### Epidemiological study of JE cases

Wuhan Children's Hospital, the only hospital accredited for diagnosing JE in Wuhan, reported all suspected JE cases of Wuhan residents during 2009 to 2010. A total of 31 cases were laboratory-confirmed JE. The serum did not have cross-reactivity to dengue virus. Eleven JE cases had disease onset in 2009 and 20 had disease onset in 2010; the incidence rates were 0.14/100,000 in 2009 and 0.25/100,000 in 2010 (chi-squared test, p>0.05). With no travel history within two weeks before onset, all JE cases went to Wuhan Children's Hospital for treatment. Most of the cases demonstrated typical encephalitic symptoms, i.e. unconsciousness, lethargy, and mental clouding. After treatment, 27 patients recovered, 3 patients presented with dementia symptoms, and 1 patient died. The prevalence of JEV was slightly higher in males than in females with a M∶ F ratio of 1.38∶1 (18/13). All patients were children between 2 months and 9 years of age with a median age of 2 years. Twenty-two (70.97%) patients lived at home, five (16.13%) were in day-care centres, and four (12.90%) were students. All of the JE cases presented onset of the disease between July and August, except for one in May. Nine patients received a JE vaccination before onset. Of these, three received two doses of the vaccine and six received one dose of the vaccine. Eleven (35.48%) cases had not been immunized, and the JE vaccination status of the remaining 11 (35.48%) cases was unknown. The details are presented in Table 1. Information regarding vaccination dates, date of onset, and outcome of the disease is included in Table S1.

**Table 1:** Characteristics of Japanese encephalitis cases in Wuhan, China (2009–2010).

Characteristic		No.	%
No. of cases		31	
Gender	Male	18	58.06
	Female	13	41.94
Age group	<8 months	3	9.68
	8 months-1 year	5	16.13
	2–5 years	17	54.84
	6–9 years	6	19.35
Living status	at home	22	70.97
	in day-care center	5	16.13
	at school	4	12.90
Immunization status	2 doses	3	9.68
	1 dose	6	19.36
	0 doses	11	35.48
	Unknown	11	35.48
Antibody results	IgM positive	31	100
	IgG positive	7	22.58
Outcome of the disease	Recovery	27	87.10
	Dementia	3	9.67
	Death	1	3.23

In 2009, JE cases occurred in four different districts of Wuhan, and 72.73% of JE patients resided in Huang Pi District, a rural area of Wuhan. In 2010, JE cases were widely distributed across nine districts of Wuhan, the patients either were children living in rural areas or were children who left their hometown and lived in a place without a local registration card (migrant children). The details are shown in Table 2.

**Table 2:** Area distribution of Japanese encephalitis cases in Wuhan, China (2009–2010).

	2009		2010		Total	
District	No.	%	No.	%	No.	%
Huang pi*	8	72.73	4	20.00	12	38.71
Xin zhou*	0	0	4	20.00	4	12.90
Hong san*	1	9.09	3	15.00	4	12.90
Jiang xia*	1	9.09	3	15.00	4	12.90
Jiang an	1	9.09	2	10.00	3	9.67
Han yang	0	0	1	5.00	1	3.23
Dong xihu*	0	0	1	5.00	1	3.23
Han nan*	0	0	1	5.00	1	3.23
Cai dian*	0	0	1	5.00	1	3.23
Total	11	100	20	100	31	100

Rural areas are indicated by an asterisk (*).

### Serological analysis for piglets

Serum samples were collected from 209 piglets at farms in Dongxihu District and detected for JEV antibody. As indicated in Table 3, the serum prevalence of JEV-specific antibodies was seasonally related; it was 100% JEV antibody positive in July and August, but it had a lower prevalence in April (66.67%) and October (37.93%).

**Table 3:** Prevalence of neutralizing antibodies to Japanese encephalitis virus among piglets in Wuhan, China, 2009.

Month	No. of samples	No. JEVAb* (+)	% JEVAb (+)
April	30	20	66.67
May	30	25	83.33
June	30	24	80.00
July	30	30	100
August	30	30	100
September	30	21	70.00
October	29	11	37.93
Total	209	161	77.03

* JEV Ab: Japanese Encephalitis Virus Antibody.

### Mosquito monitoring

A total of 84,089 adult mosquitoes were trapped nearby JE case's houses. *Culex tritaeniorhynchus* was the major type of mosquito (90.77%). Some of the mosquitoes belonged to *Culex pipiens fatigans* (4.22%) and to *Anopheles hyrcanus sinensis* (3.56%). The details are shown in Table 4.

**Table 4:** Classification of adult mosquitoes trapped nearby Japanese encephalitis cases' homes in Wuhan, China, 2009–2010.

Category	*Culex tritaenio-rhynchus*	*Culex pipiens fatigans*	*Anopheles hyrcanus sinensis*	*Armigeres obturbans*	*Culex bitaenio-rhynchus*	*Aedes vexans*	*Mansonia uniformis*	*Aedes albopictus*
No.	76327	3547	2996	853	188	159	11	8
%	90.77	4.22	3.56	1.01	0.22	0.19	0.01	0.01

### Virus identification, multiple alignments, and phylogenetic analysis

**Figure 2:**
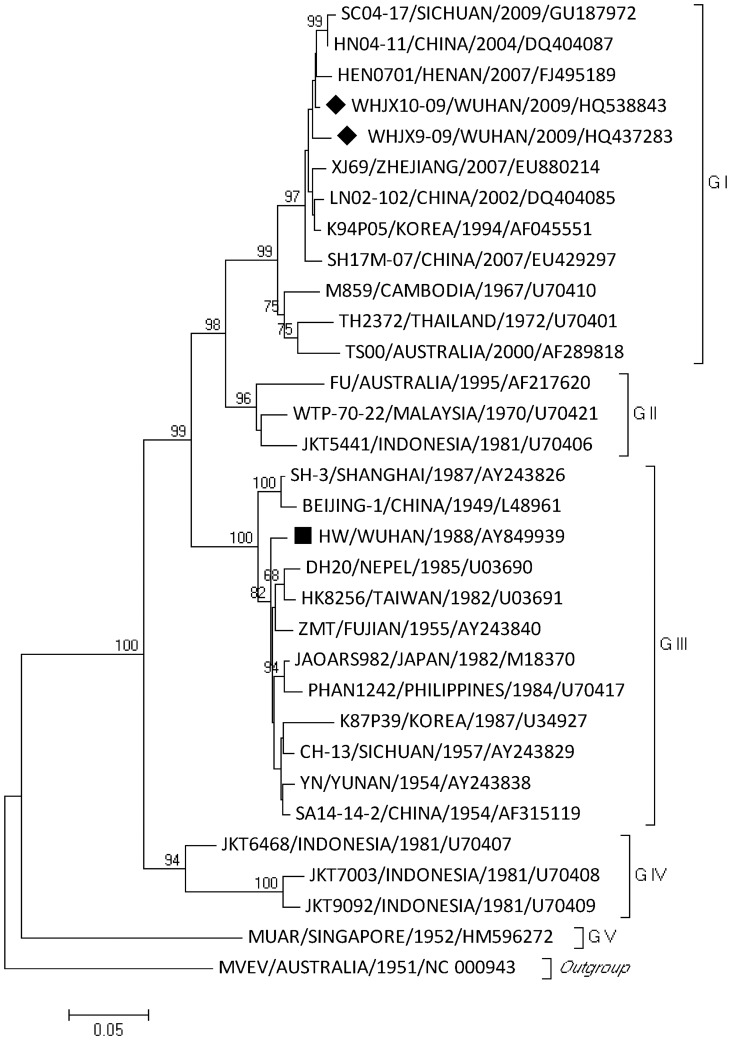
Phylogenetic tree based on the full E gene of the representative JEV strains. The tree was constructed using the neighbor-joining method in Mega software version 4.0. The scale at the bottom of the tree indicates the number of nucleotide substitutions per site. Horizontal branch lengths are proportional to the genetic distance, while vertical branch lengths have no significance. The numbers above each branch represent the percentage of 1000 bootstrap replicate support. The tree was rooted using an outgroup sequence of Murray Valley encephalitis virus (MVEV). Newly isolated JEV strains in Wuhan are labeled with black diamonds on the left and belong to genotype 1. The older strain belonging to genotype 3 is labeled with a black square.

Two new strains of the JE virus were isolated and identified from *Culex tritaeniorhynchus*. They belonged to genotype 1. No JE virus strains were isolated from other types of mosquitoes. These two new JE virus strains were named as WHJX09-09 (Genbank accession number HQ437283) and WHJX09-10 (Genbank accession number HQ538843). The phylogenetic analysis is shown in Figure 2. The homology of the two new JE virus strains was 98.90% in the nucleotide sequences and 100% in the deduced amino acid sequences. Comparing the two new JE virus strains to live attenuated vaccine strain SA14-14-2 in the E gene, the homology of the nucleotide sequence was 87.93% (WHJX09-09) and 88.33% (WHJX09-10), respectively, and the homology of amino acids was 96.90% (a total of 15 amino acids were different from SA14-14-2). The mutated amino acids were located in the E gene antigen determinants, domain I (E138, 176, and 177), domain II (E57, 107, 129, 222, 244, 264, and 279), and domain III (E315, 327, and 366). The details are shown in Table 5.

**Table 5:** Comparison of the SA14-14-2 strain with two newly isolated JEV strains, WHJX09-09 and WHJX09-10, at the amino acid level of E protein.

Strain	E56	E107	E129	E138	E176	E177	E222	E244	E264	E279	E315	E327	E366	E439	E447
SA14-14-2	Val	Phe	Thr	Lys	Val	Ala	Ala	Gly	His	Met	Val	Ser	Ala	Arg	Asp
WHJX09-09	Ile	Leu	Met	Glu	Ile	Thr	Ser	Glu	Gln	Lys	Ala	Thr	Ser	Lys	Gly
WHJX10-09	Ile	Leu	Met	Glu	Ile	Thr	Ser	Glu	Gln	Lys	Ala	Thr	Ser	Lys	Gly

## Discussion

In the present study, we reported the recurrence of JE in Wuhan, China, during 2009–2010. A total of 31 cases were laboratory-confirmed JE, 11 cases were onset in 2009, and 20 cases occurred in 2010. We investigated the patients' immunization status and analyzed the epidemiological data of piglets and mosquitoes in the areas of JE cases as well as molecular characterization of JEV strains to reveal the underlying causes of the JE epidemic in Wuhan.

Wuhan is situated in the middle of Hubei Province, China (East Longitude 113°41′–115°05′, North Latitude 29°58′–31°22′) and has a humid subtropical (Koppen Cfa) climate with abundant rainfall and four distinctive seasons. The annual average temperature is 15.8–17.5°C, and the average annual rainfall is 1269 mm. The geographical and environmental conditions are very suitable for mosquitoes to grow and reproduce. JEV is maintained in a natural cycle of transmission involving mosquitoes, wading birds, and pigs, with occasional infection of humans as dead-end hosts. Domestic pigs are the most common source of infection for mosquitoes, which then transmit JEV to humans. Culex mosquitoes, especially *Cx. tritaeniorhynchus*, are the principal vector for both zoonotic and human JEV transmission [12], [13], [24], [25], [26]. The majority of mosquitoes trapped from JE case household settings were *Cx. Tritaeniorhynchus*, and a high prevalence of antibodies to JEV was detected in piglets. All these data together indicate that the Wuhan area is still a natural epidemic focus of JE.

The immunization status of JE cases indicated that 9 of 31 cases had clear immunization history of the JE vaccine. Among these nine patients, three received two doses of vaccine and six received one dose of vaccine. Eleven cases were not immunized with the JE vaccine, and the immunization history for the remaining 11 cases was unclear. All patients who received two doses of vaccine completely recovered, one patient who only received one dose of vaccine developed dementia, two patients who were not vaccinated developed dementia, and one child who had not been vaccinated died. In addition, all patients with unclear immunization history recovered. These data indicate that JE recurrence was associated with either failure of vaccination or missed vaccination.

In Wuhan, JE live attenuated vaccine (LAV) of the SA14-14-2 virus was used for preventing disease. Studies have shown that this vaccine is safe, well tolerated, and highly immunogenic, and a single dose of JE vaccine is highly efficacious [27], [28]. In addition, a test in mice for evaluating the protective efficacy of this vaccine has shown that the protection efficacy against intraperitoneal challenge with 16 virus strains (both genotype 1 and genotype 3) was 80–100% [29]. There are two JEV strains circulating in China: genotype 3 and genotype 1. Genotype 1 JEV was first isolated from mosquitoes in Yunnan Province in 1979 [30], and it was also detected from mosquitoes in the provinces of Liaoning, Heilongjiang, Fujian, Hunan, and Shanxi [30], [31], [32]. In the Wuhan area, a genotype 3 JEV strain was isolated from mosquitoes in 1988 (Genbank accession number AY849939), and no genotype 1 JEV strain has been reported. In the present study, we identified two new JEV strains for the first time, and both of these strains belong to genotype 1.

To understand if these two new JEV strains led to the vaccine losing its protective efficacy and could be the cause of the 9 children who had received the JE vaccination and were subsequently infected with JE, we compared the homology of the JEV envelope gene between WHJX09-09, WHJX09-10, and SA14-14-2 strains and found that there were 15 mutated amino acids (3.10%) in either the WHJX09-09 or the WHJX09-10 strain. Thirteen of the15 mutants were located in the E gene antigen determinant region, which plays an important role in viral attachment, virulence, and immune response induction. JEV E138, which was mutated from Glu to Lys in SA14-14-2 for the purpose of reducing vaccine toxicity, still contained a Glu residue in both WHJX09-09 and WHJX09-10 strains, meaning that new strains of JEV would have strong viral virulence to patients. However, there were no mutations in the E protein domain III residues (E307–E309, E327–E333, and E386–E390). Previous studies have shown that these three domain III residues of E protein could form a novel cis-proline turn structure and are important in eliciting JEV-specific neutralizing antibodies [33]. Based on this information, it seems that the current vaccine still possesses the ability to protect children from JEV infection, at least in theory. The nine children who had received the JE vaccine but still acquired infection may be associated with a change in the immune response, since the mutation of new isolates in Wuhan affected the antigen determinants of the JEV envelop protein. A similar JE case was also reported from Yunnan Province, where genotype 1 JEV was obtained from the cerebrospinal fluid sample of a 2-year-old boy who had been immunized with one dose of JE live attenuated vaccine [1].

Eleven of 31 JE cases had not been immunized with the JE vaccine, and 11 cases did not have a clear immunization record, indicating that the JE vaccination program was not widespread in the JE infection area. Thus, we performed a retrospective study of the JE immunization strategy in Wuhan. Before 2008, all District CDCs implemented a booster vaccination program for susceptible children aged younger than 7 years old in the spring, before the mosquito population increased. Starting in 2008, the live attenuated JE vaccine was included in the expanded immunization program in China. As a result, local CDCs no longer carried out the mass campaign against JE. However, due to limited knowledge and information on the vaccine, caregivers in migrant populations, especially in the countryside, had little or no access to JE vaccination, leading to children losing the opportunity to be immunized. This might be the primary reason that children living in rural areas have a low JE vaccination coverage.

Due to lack of a specific antiviral treatment for JE, JE continues to be an important public health problem. Vaccination is still the most important strategy for preventing the disease. Currently in China, the JEV vaccine is included in the routine immunization protocol and is free for children. However, some caregivers do not realize the serious consequences of JE infection, and this leads to some children not being immunized, especially in rural areas. For improving the JE immunization coverage rate in poor and remote areas, the local CDC still needs to organize a mass campaign against JE, and more seminars need to be provided for caregivers to better understand JE. In addition, new JE vaccines may need to be developed against new strains of JEV.

## Supporting Information

Table S1 The status of vaccination and the outcome of the disease.
(DOC)
