# Supplementary material for: Recurrence of Japanese Encephalitis Epidemic in Wuhan, China, 2009–2010
Source: PLoS One. 2013 Jan 9;8(1):e52687. doi: 10.1371/journal.pone.0052687 (PMC3541373; doi:10.1371/journal.pone.0052687)
Supplement: Table S1 — The status of vaccination and the outcome of the disease. (DOC) [file pone.0052687.s001.doc]

| **Id** | **Sex** | **Birthday** | **Status of vaccination** | **Date of first dose** | **Date of second dose** | **Date of onset** | **Outcome** |
| --- | --- | --- | --- | --- | --- | --- | --- |
| 1 | Male | 2000-10-26 | 2 doses | 2002-3-6 | 2003-4-9 | 2010-7-25 | Recovery |
| 2 | Male | 2006-9-30 | 2 doses | 2007-6-25 | 2008-8-20 | 2010-7-28 | Recovery |
| 3 | Male | 2007-12-29 | 2 doses | 2008-6-5 | 2009-10-16 | 2010-7-20 | Recovery |
| 4 | Male | 2005-2-21 | 1 dose | 2006-9-21 |  | 2010-8-12 | Recovery |
| 5 | Male | 2006-11-29 | 1 dose | 2008-4-17 |  | 2009-7-17 | Dementia |
| 6 | Male | 2007-8-26 | 1 dose | 2009-10-13 |  | 2010-8-20 | Recovery |
| 7 | Female | 2008-2-25 | 1 dose | 2008-12-29 |  | 2009-7-8 | Recovery |
| 8 | Male | 2009-8-8 | 1 dose | 2010-5-11 |  | 2010-5-29 | Recovery |
| 9 | Male | 2009-11-13 | 1 dose | 2010-8-17 |  | 2010-8-31 | Recovery |
| 10 | Male | 2001-7-21 | 0 dose |  |  | 2010-7-16 | Recovery |
| 11 | Male | 2002-7-8 | 0 dose |  |  | 2009-7-13 | Recovery |
| 12 | Male | 2003-4-29 | 0 dose |  |  | 2010-7-20 | Dementia |
| 13 | Male | 2004-1-10 | 0 dose |  |  | 2010-8-18 | Dementia |
| 14 | Male | 2005-7-6 | 0 dose |  |  | 2009-7-19 | Recovery |
| 15 | Female | 2006-9-30 | 0 dose |  |  | 2009-7-14 | Recovery |
| 16 | Female | 2007-5-19 | 0 dose |  |  | 2009-7-15 | Death |
| 17 | Female | 2009-9-12 | 0 dose |  |  | 2010-7-17 | Recovery |
| 18 | Female | 2009-12-21 | 0 dose |  |  | 2010-8-10 | Recovery |
| 19 | Female | 2010-4-28 | 0 dose |  |  | 2010-7-20 | Recovery |
| 20 | Female | 2010-6-12 | 0 dose |  |  | 2010-8-2 | Recovery |
| 21 | Male | 2004-8-29 | Unknown |  |  | 2009-7-15 | Recovery |
| 22 | Male | 2005-7-29 | Unknown |  |  | 2010-7-30 | Recovery |
| 23 | Female | 2006-8-22 | Unknown |  |  | 2009-7-17 | Recovery |
| 24 | Female | 2006-9-14 | Unknown |  |  | 2009-7-17 | Recovery |
| 25 | Female | 2006-12-3 | Unknown |  |  | 2010-7-27 | Recovery |
| 26 | Female | 2007-1-30 | Unknown |  |  | 2010-7-25 | Recovery |
| 27 | Male | 2007-7-23 | Unknown |  |  | 2009-7-13 | Recovery |
| 28 | Male | 2007-10-28 | Unknown |  |  | 2010-8-6 | Recovery |
| 29 | Female | 2008-1-19 | Unknown |  |  | 2010-7-16 | Recovery |
| 30 | Female | 2008-1-19 | Unknown |  |  | 2009-7-6 | Recovery |
| 31 | Male | 2008-8-9 | Unknown |  |  | 2010-8-10 | Recovery |
